# Current Advances in Aptamers for Cancer Diagnosis and Therapy

**DOI:** 10.3390/cancers10010009

**Published:** 2018-01-03

**Authors:** Shin-ichiro Hori, Alberto Herrera, John J. Rossi, Jiehua Zhou

**Affiliations:** 1 Department of Molecular and Cellular Biology, Beckman Research Institute of City of Hope, 1500 E. Duarte Rd, Duarte, CA 91010, USA; shori@coh.org (S.-i.H.); albherrera@coh.org (A.H.); 2 Drug Discovery & Disease Research Laboratory, Shionogi & Co., Ltd., 3-1-1, Futaba-cho, Toyonaka, Osaka 561-0825, Japan; 3 Irell and Manella Graduate School of Biological Sciences, Beckman Research Institute of City of Hope, 1500 E. Duarte Rd, Duarte, CA 91010, USA

**Keywords:** cell-specific aptamer, SELEX, cancer diagnosis, cancer therapy, targeted delivery

## Abstract

Nucleic acid aptamers are single-stranded oligonucleotides that interact with target molecules with high affinity and specificity in unique three-dimensional structures. Aptamers are generally isolated by a simple selection process called systematic evolution of ligands by exponential enrichment (SELEX) and then can be chemically synthesized and modified. Because of their high affinity and specificity, aptamers are promising agents for biomarker discovery, as well as cancer diagnosis and therapy. In this review, we present recent progress and challenges in aptamer and SELEX technology and highlight some representative applications of aptamers in cancer therapy.

## 1. Introduction

The conventional anticancer strategies of chemotherapy and radiotherapy are highly effective at killing cancer cells, but they lack target specificity and can also kill healthy noncancerous cells [1], resulting in unwanted side effects, such as nausea and vomiting [2]. Recently, targeted cancer therapies have been designed to reduce potential toxicity and achieve a higher therapeutic index [3,4]. Therapies that use monoclonal antibodies to target tumors are the most successful cancer-targeting treatments [5,6]; however, several limitations, such as high production costs and low penetration of solid tumors, cannot be overlooked [7,8]. Thus, the development of cheaper, more effective targeted therapies is eagerly desired.

Nucleic acid aptamers are short single-stranded oligonucleotides that fold into unique three-dimensional structures and bind to a wide range of targets, including proteins [9,10], small molecules [11], metal ions [12,13,14], viruses [15], bacteria [16] and whole cells [17,18], with high specificity and binding affinities (from the low nanomolar to picomolar range) similar to those of antibodies [19]. Aptamers also have advantages compared to antibodies, such as rapid in vitro selection, cell-free chemical synthesis, low immunogenicity and superior tissue penetration because of their smaller size. Aptamers are generally isolated through in vitro selection from oligonucleotide libraries containing random sequences. After target-specific aptamers have been identified, they can be chemically synthesized, modified and optimized for clinical applications. Therefore, aptamers are promising agents for the treatment of human diseases, including cancers, infectious diseases, and heritable diseases. In this review, we discuss recent advances and challenges in the development of aptamers as agents for cancer diagnosis and therapy, with a particular focus on the past five years.

## 2. SELEX Technology

**Figure 1:**
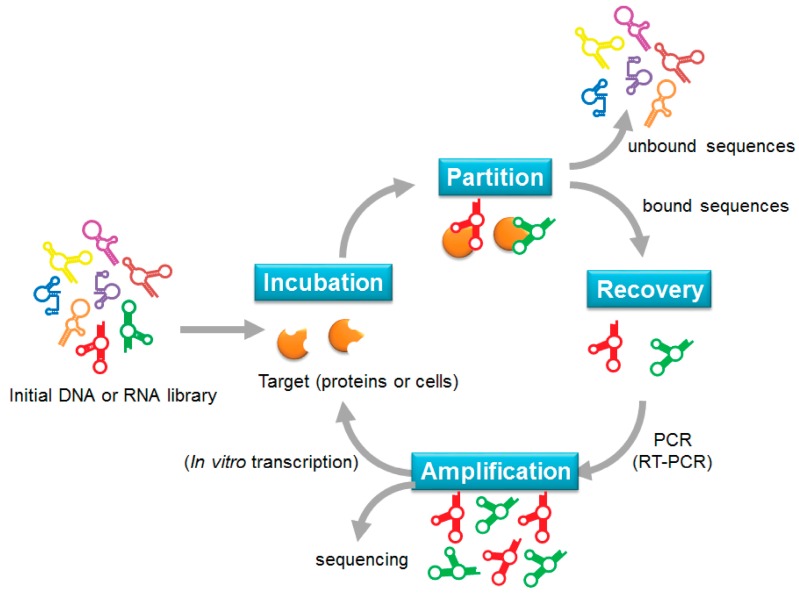
Schematic illustration of exponential enrichment (SELEX).

In the 1990s, three independent groups isolated specific RNA aptamers through a selection method called the systematic evolution of ligands by exponential enrichment (SELEX) [9,20,21]. SELEX is generally divided into four steps: incubation, partition, recovery, and amplification (Figure 1). The selection cycle starts by mixing an initial DNA or RNA library with the target of interest. A library generally consists of up to 10^15^ random sequences of 20–60-nucleotides flanked by fixed primer regions at the 5′ and 3′ ends. After incubation, target-bound sequences are separated from un-bound sequences through various partition strategies. The bound sequences are recovered and re-amplified to generate a new library for the subsequent selection cycle. New DNA libraries are directly amplified by PCR, whereas recovered RNA sequences must be reverse transcribed into cDNA before PCR amplification and transcription into a new RNA library for the next cycle. After the selection cycle has been repeated 2–15 times, sequencing analysis is used to identify the specific sequences that have been enriched in the library. To enhance the enrichment of target-bound sequences, the selection stringency can be increased during the selection cycle by manipulating the library-to-target ratio, buffer composition, incubation time, and temperature.

### 2.1. Protein-Based SELEX

Over the past 27 years, proteins have been the most common targets for aptamers. If the target proteins can be purified, protein-based SELEX can be easily performed in a test tube. One of the most critical steps in protein-based SELEX is partitioning, which involves separating target-bound sequences from unbound sequences. Various methods have been developed for partitioning, including nitrocellulose membrane filtration, affinity and magnetic bead separation, resin chromatography and capillary gel electrophoresis [22]. Although protein-based SELEX has successfully generated a wide variety of aptamers, it may be limited in several circumstances. For example, it is difficult to isolate aptamers that target unknown proteins, insoluble proteins, or proteins that have complex conformations. Furthermore, the surface of living cells is complex, and purified proteins may exist in different conformations than native proteins on the cell surface. Thus, target-specific aptamers isolated by purified protein-based SELEX may fail to recognize their target proteins on the cell surface [23,24].

### 2.2. Whole-Cell-Based SELEX

Whole-cell-based SELEX was developed to overcome the limitations of protein-based SELEX [25,26]. In whole-cell-based SELEX, live cells that express the target of interest are used instead of purified protein, enabling the identification of aptamers that can recognize targets in their native conformation. Because protein purification is not necessary prior to selection, whole-cell-SELEX can be applied to uncharacterized target proteins without prior information about their properties and structures [18,27]. Although whole-cell-based SELEX involves the same major steps as conventional protein-based SELEX, whole-cell-based SELEX requires both positive and counter selection in target-positive and target-negative cells, respectively. Counter selection is crucial for removing non-specific binders. After positive and counter selection, aptamers are expected to bind to the cells that express the target but not to the cells that do not express the target. To efficiently enrich target-specific aptamers, the cells used must be healthy. The presence of dead cells can result in enrichment of non-specific binders, delaying the enrichment of target-specific sequences. Therefore, careful recovery of healthy cells that highly express the active target is crucial for successful selection. Some technical approaches, such as fluorescence-activated cell sorting (FACS) [28] and magnetic bead separation [29], have been used to eliminate the risk of non-specific binding to dead cells, optimizing the selection and improving the generation of target-specific aptamers.

### 2.3. Live-Animal-Based SELEX

Live animals have also been used to directly generate tissue-targeting aptamers in vivo via live-animal-based SELEX [30], or in vivo SELEX [22]. Unlike whole-cell-based SELEX, counter selection is not needed for live-animal-based SELEX. In 2010, Mi et al. generated tumor-targeting aptamers by live-animal-based SELEX in intrahepatic tumor-bearing mice [31]. The authors intravenously injected a random 2′-F-pyrimidine-modified RNA library into mice with intrahepatic colorectal metastases and harvested the tumor-containing liver tissue. The target-bound RNA sequences were extracted, amplified, and used for the next selection cycle. After 14 cycles, the authors isolated RNA aptamers that specifically localized to intrahepatic tumors, one of which bound to RNA helicase p68 that is overexpressed in colorectal cancers. This report demonstrated that live-animal-based SELEX can directly generate aptamers that can be efficiently delivered into tumor tissues in vivo.

### 2.4. High-Throughput SELEX

Efforts have been made to improve the selection efficiency of SELEX [22,32]. To isolate high-affinity aptamers from random sequences, it is important to ensure a diverse library during selection and to avoid technical bias. In traditional SELEX, the multiple cycles of conventional PCR may accumulate nonspecific byproducts, causing some bias during amplification [33]. For example, some sequences favored by DNA polymerase may be over-enriched during PCR. In contrast, highly structured sequences that are difficult to amplify may eventually be eliminated. Amplification has been improved with novel PCR technologies, such as droplet digital PCR [34] and emulsion PCR [35,36,37], which can reduce the accumulation of byproducts and avoid PCR bias, thus preserving library diversity.

After amplification, the PCR products in the final library are generally cloned into *E. coli* for sequence identification. However, this step is time-consuming and laborious, and the resulting clones are not necessarily representative of the whole population of aptamers. Some infrequent, high-affinity aptamers may be missed due to limited clone number or inefficient cloning. This kind of cloning bias can be avoided by using high-throughput sequencing technology and bioinformatics analysis combined with SELEX (HT-SELEX). HT-SELEX enables visualization of dynamic changes among millions of sequence reads throughout selection, so it is possible to reduce cloning bias and identify high-affinity aptamers during a much earlier selection round. Thus, HT-SELEX can not only save money and time, but also reduce the risk of technical biases.

## 3. Recent Progress in Aptamer-Based Biosensor Technology

Biosensors are analytical devices that can measure the concentration of organic or inorganic targets, called analytes, by generating signals proportional to the analyte. Biosensors are generally composed of four parts: a bioreceptor that detects the analyte, a transducer that converts recognition of the target into a measurable signal, electronics that amplify and the signal, and a display that presents the results to the user [38]. The high specificity of aptamers makes them ideal bioreceptors in aptamer-based biosensors called aptasensors. Aptasensors are superior to antibody-based sensors because of their high affinity and stability, highly modifiable kinetic parameters, relatively fast animal-free development and wide spectrum of targets ranging from small chemicals to whole cells [39]. In addition, aptamers change conformation upon binding, and sensors have been developed that exploit this property for target detection [40]. Aptasensors have the potential for a variety of applications, including detection of foodborne pathogens, chemicals, and disease markers [38]. Several electrochemical, optical, and colorimetric aptasensor methods exist for the detection of cancer. In this section, we will focus on recent advances in aptasensors for cancer detection, with an emphasis on advances from the past year.

### 3.1. Electrochemical Aptasensors

**Figure 2:**
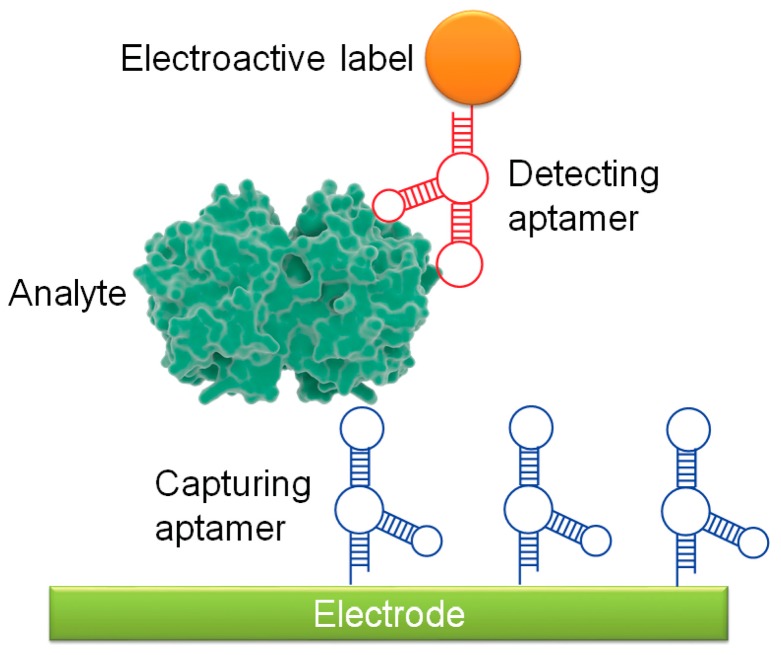
Schematic of sandwich-style electrochemical aptasensor.

One of the most common aptasensors is the electrochemical aptasensor. Electrochemical aptasensors have existed since 2004, when Ikebukuro et al. developed a sandwich-style aptasensor to detect the clotting factor thrombin [41]. A simple aptamer sandwich detection system is composed of two aptamers and an electrode surface (Figure 2). A capturing aptamer conjugated to an electrode surface captures and immobilizes the analyte, and a secondary aptamer, which recognizes a different part of the analyte surface, binds to form an aptamer-analyte-aptamer sandwich. The secondary aptamer contains an electroactive label, such as glucose dehydrogenase [41], cadmium sulfite quantum dots [42], or gold nanoparticles (AuNPs) [43], which can be detected by the electrode [38]. Because of their relative simplicity, a number of sandwich-based detection systems have been developed against cancer targets. As an example from the last year, Zhang et al. developed an electrochemical aptasensor using an aptamer against mucin 1 (MUC1), a surface glycan that is highly overexpressed in many cancers. MUC1-expressing cells were bound by MUC1 aptamer conjugated to magnetic beads, followed by capture by a secondary lectin-based nanoprobe functionalized on AuNPs [44]. In this experiment, gold-promoted reduction of silver ions induced voltage changes that, when read through electrochemical stripping analysis, were indicative of MUC1 expression levels, and thus potentially of cancer detection. Additional sandwich-style aptasensor systems targeting cancer markers and cancer cell lines are summarized in Table 1.

Label-free electrochemical aptamers have been developed that take advantage of aptamer features, including their conformational change upon target binding, increased resistance caused by double-stranded DNA formation, and decreased signaling when aptamer binding displaces an electroactive group on an electrode [38,45]. Several label-free aptamers have been developed against cancer targets especially aptasensors that take advantage of conformational change when aptamers bind to their target (Table 1). Khoshfetrat et al., for example, developed an aptasensor against leukemia cells utilizing the sgc8c aptamers that target protein tyrosine kinase 7 (PTK7), which is highly expressed in the acute lymphoblastic leukemia cell line CCRF-CEM [46]. To provide a signal, ethidium bromide (EB) was introduced and allowed to intercalate into the stem of the aptamer hairpin. When the target (i.e., PTK7) bound to the aptamer, the hairpin structure of the aptamer was disrupted, releasing the intercalating EB and decreasing the electrical signal on a nitrogen-doped graphene nanosheet that was used as an electrode surface [47]. Conformational changes upon aptamer binding can also alter the electronic transfer distance between an aptamer and an electrode. This property has been exploited through the use of modified electroactive aptamers and modified electrodes. For example, Wang et al. used a polyadenine modified aptamer system to detect MCF-7 breast cancer cells via the voltage drop recognized by differential pulse voltammetry upon target binding [48]. Heydari-Bafooei and Shamszadeh instead modified an electrode by combing reduced graphene, multi-walled carbon nanotubes, and AuNPs, to detect voltage changes based on the conformational changes of aptamers bound to the prostate cancer marker PSA [49]. Additional label-free systems targeting cancer markers and cancer cell lines summarized in Table 1.

Current efforts aim to develop low-cost, portable aptasensor platforms. Microfluidic paper-based analytical devices (µ-PADs) are one potential solution. µ-PADs use grooved hydrophilic paper containing a series of millimeter-sized channels bound by a hydrophobic polymer [78]. Detectors, including metal ions and colorimetric dyes, bound within these grooves, can generate signals from microscopic samples [79,80]. Ma et al. used a metal ion-based µ-PAD detection system to detect both carcinoembryonic antigen (CEA) and MUC1 [81]. In this electronic aptasensor, aptamers against CEA and MUC1 were bound to thiolated complementary capture probes on the surface of a µ-PAD. Upon exposure to the target, aptamers preferentially unbound from the capture probes and bound to the target. This allowed metal ion-incorporating nanospheres to bind to unoccupied capture probes though their conjugated auxiliary sequences. Conductivity was then measured through Ru(NH_3_)_6_^3+^ electronic wires [81]. The development of µ-PAD-based aptasensors for clinical use is promising, as the grooved paper can be produced using a series of low cost techniques, including wax printing, laser treating, and photolithography, which are increasingly cost-effective and scalable for mass production [78].

### 3.2. Fluorescent Aptasensors

In 1996, a fluorescent aptamer against human neutrophil elastase (HNE) was the first aptamer-based biosensor developed. The HNE fluorescent aptamer was found to be as effective as an antibody at detecting HNE on beads, with the added benefits of faster chemical synthesis and the ability to add functional groups, small size to ease internalization, potential for to detect intracellular targets, less off-target binding, and greater storage stability [82]. Since then, several fluorescent aptasensors have been developed for cancer detection. In 2006, Herr et al. developed a fluorescent sandwich system using aptamers conjugated to fluorescent nanoparticles to detect cancer cells. They used magnetic nanoparticles to facilitate extraction of these cells from whole blood samples, providing an effective system for clinical use [83]. In 2009, Chen et al. developed a multiplexed detection system to detect multiple cancer cell targets using aptamer-conjugated Forster resonance energy transfer (FRET) silica nanoparticles [84].

Fluorescent aptasensors can be used to detect not only cancerous cells but cancer markers. One such marker, vascular endothelial growth factor (VEGF), was an early target for detection by fluorescent aptamers. In 2012, Freeman et al. presented a series of optical aptasensor methods based on the conformational change of the anti-VEGF aptamer upon binding to its target. FRET-, chemiluminescence-, and chemiluminescence resonance energy transfer (CRET)-based strategies were used for target visualization [85]. Cho et al. developed a single-step detection method for VEGF165 based on nanoplasmonic sensing, the optical phenomenon in which the intensity of a fluorophore changes when it interacts with the free electrons on the surface of a metal [86]. For this strategy, cyanine (Cy3)-labeled anti-VEGF aptamers were recruited to the surface of AuNPs. Upon binding with VEGF, the aptamers changed conformation, which released them from the AuNPs, causing a significant decrease in fluorescence intensity [86].

Several studies have used FRET aptasensors to detect cancer markers. Hamd-Ghadareh et al. developed an aptamer-based system to detect CA125, a marker of several cancer types, including ovarian cancer on which this aptamer system was tested. This study used aptamer-carbon dot probes to detect CA125-positive cells and measured the FRET signals caused by the interaction of the carbon dots and AuNPs, which acted as nanoquenchers [87]. Xiao et al. used graphene-oxide (GO), which binds to single-stranded DNA, as a FRET quencher in a similar manner [88]. When the analyte was not present, fluorescent labels on the aptamer were brought in close proximity to GO and the signal was quenched, providing very low background signal. Upon contact with the analyte, the fluorescent labels were separated from the quencher and could emit a signal that was proportional to the concentration of the analyte [89].

One emerging trend is the use of nanogels for aptamer delivery and cancer detection. Iwasaki et al. created 2-methacryloyloxyethyl phosphorylcholine (MPC) nanospheres that incorporated anti-thrombin aptamers during synthesis. Two types of MPC polymers were synthesized independently, one with MPC conjugated to the aptamer and another with MPC conjugated to a strand of DNA complementary to the aptamer strand. The strands combined and self-organized into aptamer-carrying nanospheres. These nanospheres were also able to incorporate fluorescent markers, such as EB, for highly specific detection of the target, which in this case was the cancer marker thrombin [90]. Hu et al. created a simple fluorescent aptamer detection system using the biotin-conjugated aptamer TLS11a against liver cancer cells and streptavidin-conjugated fluorescein isothiocyanate (FITC)-doped silica nanoparticles. The biotin-conjugated aptamers outperformed simpler FITC conjugated nanoparticles (~90% vs. ~60%) in detection of HepG2 cells via flow cytometry [91]. Shangguan et al. also used TLS11a to make an “activatable” aptamer-based fluorescent probe [92]. In their study, a short 5′ strand was added to the fluorescent tag FAM and a complimentary 3′ C-strand was conjugated to the fluorescent quencher Eclipse. The quencher prevented fluorophore expression unless the aptamer came into contact with its target, allowing a conformational change that separated the quencher from the fluorophore [93]. The activatable aptamer approach was taken further by Lei et al., who developed a theranostic method that not only activates a fluorescent signal upon interaction with its target, but induces the release of a drug. In this proof-of-concept study, the conformational change of an aptamer against CCRF-CEM leukemia cells activated a fluorescent probe and released the chemotherapy drug doxorubicin (Dox) for cancer cell-specific drug delivery [94].

### 3.3. Colorimetric Aptasensors

Colorimetric aptasensor assays allow for simple, fast detection of targets ranging from small metal ions [95] to proteins [96]. Several aptamer-based colorimetric assays have been developed for cancer marker detection. Xu et al. targeted the proto-oncogene *K-Ras* with a colorimetric biosensing system based on a DNA molecular machine [97]. The core of the machine was a hairpin probe that targeted K-Ras and hybridized with a primer-contained polymerization template (PPT) that generated an anti-hemin aptamer. The anti-hemin aptamer activated a DNAzyme that mimicked the action of horseradish peroxidase, catalyzing the activation of the substrate 2,2′-azino-bis(3-ethylbenzothiozoline-6-sulfonic acid) (ABTS) and changing the color of the substrate from colorless to green, detectable by the naked eye [97]. A nanoparticle-based colorimetric aptasensor system was developed by Ahirwar et al. for detecting the human estrogen receptor alpha (ERα), a common marker in breast cancer [98]. The system uses the color-changing properties of gold nanospheres, which interact with light and reflect different colors based on nanosphere size and dispersion rate. Monodispersed gold nanospheres reflect red light and aggregated nanospheres reflect a pale to purple color [99,100]. The aptamer-functionalized gold nanospheres were resistant to salt-induced aggregation until they were exposed to their target, ERα, which caused spontaneous aggregation of the nanospheres, changing the color of the nanosphere clusters from a wine red to a deep blue and allowing visual identification [98].

### 3.4. Aptasensors in Clinical Diagnostics

Aptasensors provide a number of advantages for clinical diagnostics including high specificity and selectivity, and relatively low cost of production [45]. When compared to traditional antibody-based detection platforms, nucleic acid aptamers are more stable, highly modifiable, and are capable of fast animal-free development against a wide spectrum of targets ranging from small chemicals to whole cells [39]. One main disadavantage of aptasensors, which is shared with their antibody based counterparts, is that they can only detect previously known markers. This could be resolved with significant work into biomarker discovery with an emphasis on identifying biomarkers that are common across multiple cancer types. An emphasis on early cancer and metastasis markers would also be useful in tracking and early detection of cancer in high risk patients. Patients with known BRCA mutations who are prone to ovarian and breast cancer [101] and patients with Cowden Syndrome who are more at risk for breast, thyroid, and uterine cancers [102], for example, may prove ideal for aptasensor testing. Another limitation arises from the fact that each aptasensor is highly specific to one marker or cell type. Multiplexed aptasensors that detect a panel of cancer markers could be a solution, but a larger number of aptamers must first be developed. Cai et al. pointed out that many aptasensors are optimized for buffer solutions and may not be effective in biological fluids [103], but an increasing number of aptasensors have been shown to be effective in detecting markers in serum [47,49,74] signifying that this problem is a design problem not a technological one.

Despite these limitations, aptasensors provide a great opportunity for advances in clinical diagnostics. In cancer, the high sensitivity of aptamers would allow for low-cost and non-invasive cancer testing by detecting minuscule biomarker levels in the blood, urine, or other bodily fluids [104]. A wide range of aptasensors have been developed with sensitivities within an ideal clinical range and have potential for commercial use as has been summarized in Table 1 for electrochemical aptasensors and a number of aptasensor reviews [45,104,105]. Despite this, aptasensors have not yet broken into the field of clinical diagnostics, a field still dominated by immunoassays [104]. However, aptasensors are beginning to enter the market. In 2016 Jung et al. developed an aptamer-based protein biomarker panel to detect non-small cell lung cancer with 100 lung cancer and 100 control participants from the Asan Medical Center in Seoul, Korea [106]. In March of 2017 Aptamer Sciences introduced the panel commercially. According to a report by Grand View Research a number of companies including Aptamer Group (Heslington, UK); Aptamer Sciences, Inc. (Pohang, South Korea); Aptagen, LLC (Jacobus, PA, USA); Base Pair Biotechnologies, Inc. (Pearland, TX, USA).; Ophthotech Corporation (New York, NY, USA); are poised to develop aptamer-based diagnostic or therapeutics taking up a market share estimated to reach 8.91 billion by 2025 [107]. While slow to adapt as of now, with advances in aptasensor technology, cancer, biomarker discover, and aptamer development, aptasensors pose a significant threat to traditional immunoassays as the clinical diagnostics of choice.

## 4. Development of Cancer-Specific Aptamers for Diagnosis

Aptamers have been used to detect a variety of cancers by targeting tumor markers, such as nucleolin [108], tenascin [109], prostate-specific membrane antigen (PSMA) [110], MUC1 [111,112], annexin A2 [113], and matrix metalloprotease-9 (MMP-9) [114,115]. In this section, we will highlight advances in aptamer design for the detection of early, metastatic, and multiple cancers, with an emphasis on reports from the past year.

### 4.1. Aptamers for Early Cancer Detection

Early cancer detection drastically increases survival rates and treatment options [116]. Aptamer-based cancer detection systems may enable earlier, more sensitive cancer detection because they are highly specific and only require small quantities of analytes to generate signals. Lung cancer would benefit from early diagnosis because it often is not detected until it has progressed to a late stage, when five-year survival rates approach single digits [117,118]. Li et al. isolated six DNA aptamers against lung cancer markers using a modified SELEX technique involving magnetic carboxyl agar beads [118]. During this process, the beads were incubated with clarified mixed serum from healthy individuals for negative selection, followed by positive selection by beads incubated with serum from lung cancer patients. These six aptamers were shown to be highly specific in detecting lung cancer in the serum of 20 lung cancer patients but not of 20 healthy patients. This system was much more sensitive than traditional lung cancer diagnosis methods, thus potentially enabling earlier diagnosis [118].

Gynecological cancers are also difficult to diagnose at an early stage. Tsai et al. used an aptamer-based microfluidic system to capture and detect circulating tumor cells (CTCs), which are generally found at extremely low concentrations and circulate irregularly. By using highly specific aptamers, the system allows for a high rate of CTC discovery, low false positives, and quick detection when compared to antibody-based detection of ovarian cancer [119].

### 4.2. Aptamers Targeting Metastatic Cancer

Patient outcomes can also be improved by detecting metastasis. One of the most common ways of developing aptamers to detect metastatic cells is to perform SELEX between metastatic and non-metastatic variants of established cancer lines. Yuan et al. isolated an aptamer for metastatic colorectal cancer via SELEX by using metastatic colorectal carcinoma LoVo cells for positive selection and non-metastatic colorectal carcinoma SW480 and HT-29 cells for negative selection [120]. The aptamer, fluorescently labeled with cyanine (Cy5), recognized colorectal carcinoma metastases in lymph node tissue with a detection rate of 73.9% and low detection of non-metastatic carcinoma (36.7%) and cancer-adjacent tissues (11.1%) [120]. Duan et al. developed an aptamer, DML-7, for metastatic prostate cancer by using SELEX with the metastatic prostate cancer line DU145 for positive selection and the human prostatic stromal myofibroblast line WPMY-1 for counter selection [121]. This aptamer was then tested on cells that were androgen receptor (AR)-negative (PC3) and AR-positive (LNCaP and 22Rv1), because ARs may suppress metastatic potential and are often upregulated in patients with metastatic prostate cancer [121,122]. The authors found that DML-7 bound to AR-negative PC-3 cells but not AR-positive LNCaP and 22Rv1 cells. However, the authors could not identify the receptor to which the aptamer bound and also found that it did not bind exclusively to metastatic prostate cells. The aptamer was found to bind to the adenocarcinomal cell line PL45, the human lung adenocarcinoma line A549, and the osteosarcoma cell line U2OS [121]. For better specificity to highly metastatic cells, Chen et al. developed aptamers against hepatocellular carcinoma cells from high metastatic (HCCLM9) and low metastatic (MHCC97L) cell lines derived from the same genetic background. These aptamers were found to have high specificity to the high metastatic line but did not bind to any of the other cell lines tested suggesting high specificity to HCCLM9 [123,124].

A more direct approach for detecting metastasis is to directly target metastasis-associated proteins. An RNA aptamer created by Kryza et al. targets MMP-9 [114], which is overexpressed in tumors and promotes metastasis by degrading the extracellular matrix to facilitate tumor cell invasiveness [125]. The RNA MMP-9 aptamer, F3B, was developed via SELEX against purified hMMP-9 protein using a 2′-F-pyrimidine-modified initial RNA library to make the RNA aptamer resistant to RNAse [115]. In the study, the authors created the radiolabeled constructs ^99m^Tc-MAG-F3B and ^111^In-DOTA-F3B. Biodistribution studies showed that ^99m^Tc-MAG-F3B detected hMMP-9 in mice with A375 melanoma tumors but had high accumulation in the digestive tract. The ^111^In-DOTA-F3B construct was found to have higher tumor uptake but high accumulation in the kidneys and bladder and low uptake into the digestive tract [114]. MacDonald et al. created a bispecific aptamer targeting epithelial cell adhesion molecule (EpCAM) and transferrin to specifically target brain cancer metastases. The bispecific aptamer had higher binding to metastatic brain cancer cells than EpCAM and transferrin aptamers alone. The aptamers had the added benefit of being able to pass through the blood-brain barrier, an obstacle that often hinders the treatment of brain disorders [126].

Another innovative way to detect metastasis is to identify morphological changes in captured cancer cells. Mansur et al. captured metastatic (MDA-MDB-231) and non-metastatic (MCF-7) breast cancer cells with anti-epidermal growth factor receptor (EGFR) aptamers on plane and nanotextured substrates, which exaggerate the morphological characteristics of the cells. The authors compared the shapes and sizes of cells captured and found that metastatic cells had a significantly greater change in morphology between nanotextured and plane substrates than did non-metastatic cells [127].

CTCs are also a common target for the detection of metastatic cancers. Several groups have developed methods to detect rare CTCs using AuNPs and plasma mass spectrometry [128], aptamer-linked magnetic particles, aptamer-functionalized microchannel/microstructures [129], and a label-free electrochemical cytosensor targeting overexpressed EpCAM, which is common in CTCs.

### 4.3. Aptamers Targeting Multiple Cancers

MUC1 is a glycoprotein that is overexpressed on the cell surface of most malignant epithelial cancers, including colorectal [130], lung [131], prostate [132], pancreatic [133], ovarian [134], and bladder cancers [135]. It is overexpressed in 0.9 million of the 1.2 million cancers diagnosed in the United States each year [136]. In normal cells, MUC1 provides a barrier between the cells and the environment. In cancer cells, its overexpression enhances invasiveness, metastasis, and resistance to reactive oxygen species [137]. Because of its ubiquity and high expression in cancer, MUC1 is an excellent target for multiple cancers. Several techniques for detecting MUC1 have been in development over the last two decades, including an electrochemical aptasensor (discussed in Section 3.1) [44], a fluorescent aptasensor using GO-based fluorescent quenching (discussed in Section 3.2) [89], an antibody-based nanowire sensor [138] and an aptamer-quantum dot-based detection method [139].

Ma et al. developed a dual-targeting electrochemical aptasensor to simultaneously detect CEA and MUC1 in multiple cancers (discussed in Section 3.1) [81]. As described in Section 3.1, Zhang et al. developed a sandwich-type electrochemical aptasensor to capture MUC1-overexpressing MCF-7 human breast adenocarcinoma cells [44]. This aptasensor system also enabled colorimetric assessment by catalyzing the deposition of silver for naked-eye detection of MCF-7 cells [44]. Santos do Carmo et al. used a technetium-99m-labeled silica-based polymeric nanoparticle loaded with anti-MUC1 aptamers to deliver drugs and radiolabel triple negative breast cancer (TNBC). Biodistribution studies showed that the nanoparticle-aptamer construct was highly absorbed by the intestine (30%), but was also taken up by the tumor (5%), which is a high rate for targeted drug delivery [140]. In contrast to the use of silica nanoparticles, Yu et al. successfully used MUC1 aptamers to deliver anti-cancer paclitaxel-loaded liposomal formulations to MCF-7 cells [141]. One issue raised by Cao et al. is that many of the above-mentioned strategies do not easily detect low-expressing, low-abundance protein biomarkers and require florescent probe modifications or complicated procedures to develop and use these aptasensors [142]. To address these issues, Cao et al. developed a MUC1 detection system using immuno loop-mediated isothermal amplification (Im-LAMP), which uses Bst DNA polymerase and a group of specialized primers for highly selective DNA amplification under isothermal conditions [143]. Their aptamer-based Im-LAMP system consisted of a MUC1 aptamer to capture the target protein, followed by LAMP amplification of the targeting aptamer and measurement via real-time fluorescent PCR [143].

Cancer/testis antigens (CTAgs) are also potential targets for multiple cancers. CTAgs are a group of proteins that are generally only expressed in the immune-privileged testes of adult males. However, CTAgs are highly overexpressed in many cancers, including bladder, prostate, non-small cell lung carcinomas, and melanoma [144]. Several CTAgs, including the melanoma-associated antigen family, synovial sarcoma X antigens, and the immunogenic tumor antigen NY-ESO-1, have been identified as candidates for adaptive immunotherapies and cancer vaccines [145,146]. CTAgs are promising targets for aptamer-based detection of cancer; however, several challenges may slow the development of CTAg-targeted aptamers. One potential concern is that more than 90% of CTAgs are predicted to be intrinsically disordered proteins, meaning they are biologically active but lack a rigid 3D structure [147]. Most CTAgs, however, transition to a rigidly structured shape upon binding to a target, which may make aptamer detection possible [147,148]. Additionally, CTAgs have many alternative splicing forms and post-translational modifications [149,150], which increase their diversity and functional variability but provide a challenge for CTAg aptamer development. As of yet, no aptamers against CTAgs have been developed, but several clinical trials testing cancer vaccines that sensitize the immune system to CTAg-expressing cancer cells are in development [146]. If the obstacles to anti-CTAg aptamer development are overcome, CTAgs may prove useful for aptamer-directed identification and treatment of multiple cancers. 

## 5. Application of Aptamers in Cancer Therapy

Aptamer-based cancer therapies can be divided into two major types: (1) target antagonists and (2) delivery vehicles for therapeutic agents. In this section, we focus on aptamers that directly antagonize their pro-cancer targets. To date, two antagonistic aptamers have been evaluated in clinical trials for cancer treatment (Table 2). Here, we summarize these clinical trials and highlight recent preclinical studies of several more therapeutic aptamers for cancer treatment.

### 5.1. Clinical Trials of Cancer-Targeting Aptamers

AS1411 is a 26-nt guanosine-rich G-quadruplex DNA oligonucleotide developed by Antisoma that was the first aptamer to enter clinical trials for cancer treatment. AS1411 was not isolated by SELEX, but instead was discovered in a screen for antiproliferative DNA oligonucleotides [151]. AS1411 shows high affinity for the external domain of nucleolin, which is expressed in the nuclei of all cells, overexpressed on the surfaces of tumor cells, and involved in cell survival, growth and proliferation [152]. After binding to nucleolin, AS1411 is efficiently internalized, even at nanomolar doses [153]. AS1411 inhibits the function of nucleolin in cancer cells and shows great antiproliferative activity in various types of cancers, including lung, prostate, breast, cervical, and colon cancers, as well as malignant melanoma and leukemia. In a phase I clinical trial, AS1411 delivered by continuous infusion at doses up to 40 mg/kg/day specifically inhibited nucleolin without causing serious side effects in a variety of tumor types (ClinicalTrials.gov identifier NCT00881244). In a 2009 phase II clinical trial, AS1411 safely and effectively treated patients with primary refractory or relapsed acute myeloid leukemia (AML) (ClinicalTrials.gov identifier NCT00512083). However, in a subsequent phase II trial for renal cell carcinoma, only one of 35 patients had a response to AS1411 treatment (ClinicalTrials.gov identifier NCT00740441) [154]. Although the underlying mechanism of AS1411 action is not fully understood, the patient who showed a response to AS1411 had mutations in fibroblast growth factor receptor 2 (FGFR2) and mechanistic target of rapamycin (mTOR), suggesting potential pathways and predictive biomarkers for AS1411 treatment.

NOX-A12 is a 45-nt L-ribose-based RNA aptamer, known as a Spiegelmer, developed by NOXXON Pharma AG. Spiegelmers are mirror-image oligonucleotides that have high resistance to nucleases [155,156]. NOX-A12 was developed against chemokine C-X-C motif ligand 12 (CXCL12; also known as stromal cell-derived factor-1) and linked to a 40-kDa polyethylene glycol to give it a longer half-life in plasma [157]. CXCL12 binds to CXCR4 and CXCR7 chemokine receptors, which have important roles in tumor proliferation, metastasis, and angiogenesis, as well as regulation of leukemia stem cell migration [158,159]. Because CXCL12/CXCR4/CXCR7chemokine axis activation regulates the pattern of tumor growth and metastatic spread to organs expressing high levels of CXCL12, NOX-A12 was expected to be useful in the treatment of several types of cancers, including multiple myeloma, lung, colorectal and brain cancers [159]. In phase I clinical trials, NOX-A12 was well tolerated (ClinicalTrials.gov identifiers NCT00976378 and NCT01194934). Currently, NOX-A12 is being studied in phase II clinical trials for the treatment of chronic lymphocytic leukemia (ClinicalTrials.gov identifier NCT01486797), relapsed multiple myeloma (ClinicalTrials.gov identifier NCT01521533), and metastatic pancreatic cancer (ClinicalTrials.gov identifier NCT03168139).

### 5.2. Recent Progress in Therapeutic Aptamers for Cancer Therapy

Aptamers have several advantages over current cancer therapies, such as chemotherapies and monoclonal antibodies. Compared to monoclonal antibodies, aptamers have similar target binding affinity and specificity but also several advantages, such as rapid in vitro selection, low immunogenicity, and superior penetration into solid tumor tissue. Because aptamers are obtained by chemical synthesis, they can be easily modified, and their production costs may be lower than those of monoclonal antibodies. Therefore, aptamers have vast potential for therapeutic use. However, aptamers still have several crucial limitations, such as short in vivo duration due to nuclease-mediated degradation and rapid renal filtration, a lack of comprehensive toxicity studies, and some exclusive patents that limit the global distribution of aptamer technology. Despite these challenges, several aptamers have been reported as potential candidates for cancer therapy [160], and recent progress in SELEX technology and lessons learned from the preceding clinical trials are expected to enhance translation to the clinic. In this section, we highlight several studies published in the past five years, which investigate aptamers that show anticancer activity in vivo.

Human epidermal growth factor receptor 2 (HER2/ErbB2) is a member of the EGFR family and is overexpressed in various types of cancer, including breast and gastric cancers [161]. Recently, Mahlknecht et al. developed a DNA aptamer targeting HER2 [162]. The HER2 targeting aptamer was generated by SELEX using HER2-specific polyclonal antiserum, extracts of gastric cancer cells and random PCR deletion. After an original 14-nt aptamer was isolated, it was trimerized to improve binding affinity to the target protein. The trimeric (42-nt) aptamer efficiently bound HER2-positive cells, induced internalization and lysosomal degradation of the target protein, and inhibited cancer cell growth. Furthermore, intraperitoneal administration of the trimeric aptamer reduced tumor volume in HER-2-positive cancer xenograft mice. Once highly specific, high affinity monomeric aptamers are obtained, they can be easily multimerized. The multimerization strategy may be expanded to other aptamers to increase their efficacy as therapeutic agents.

PSMA is a transmembrane protein that is primarily expressed in prostate tissue and prostate cancers [163]. Because prostate cancer cells overexpress PSMA on the cell surface, it is a promising marker for diagnosis and targeted therapy of prostate cancers [164,165]. Several reports also suggest that PSMA has enzymatic activity related to cancer progression [166,167,168]. Dassie et al. demonstrated that an RNA aptamer targeting PSMA (A9g; 43-nt) inhibits the enzymatic activity of PSMA, reducing prostate cancer cell migration and invasiveness in vitro [169]. When systemically administered in a mouse model of metastatic prostate cancer, A9g selectively targeted PSMA-positive tumors and greatly reduced metastasis without causing significant toxicity. Thus, this aptamer may be used not only as a direct antagonist, but as a dual inhibitor via aptamer–drug conjugates targeting PSMA-positive tumors.

Programmed death 1 (PD-1) is an immune checkpoint protein expressed on the surface of T cells that functions as a negative regulator of immune responses [170]. Using protein-based SELEX, Prodeus et al. isolated a 75-nt PD-1-targeting DNA aptamer, MP7 [171]. MP7 recognized the extracellular region of PD-1, bound to murine PD-1 with nanomolar affinity, blocked the interaction between PD-1 and programmed death-ligand 1 (PD-L1), and inhibited the suppression of interleukin-2 (IL-2) secretion, which is related to immunosuppression in primary T-cells. PEGylated MP7 reduced tumor growth in mice bearing colon cancer xenografts. However, because MP7 only showed specific binding to murine PD-1 not human PD-1, a human PD-1-targeting aptamer would first need to be developed for use in clinical cancer therapy.

Rong et al. reported a DNA aptamer, LY-1, that specifically binds targets on the surface of highly metastatic hepatocellular carcinoma (HCC) [124]. They isolated this aptamer by whole-cell SELEX using two cell lines that have the same genetic background but different metastatic potential, HCCLM9 and MHCC97L (mentioned in Section 4.2). Although the direct target of this aptamer is unknown, LY-1 recognized metastatic HCC with a dissociation constant (K_d_) of 167 nM, reduced migration and invasiveness of these cells in vitro, and inhibited tumor growth when administered intraperitoneally to an HCCLM9 mouse xenograft model. This aptamer may not only be a therapeutic candidate but also a molecular probe for metastatic HCC therapy.

## 6. Current Use of Aptamers as Cancer-Targeting Agents

Targeted cancer therapy is a potential strategy to lower side effects and enhance the efficacy of anticancer agents. Because aptamers bind to their targets with high affinity and specificity and are effectively internalized into cells, cancer cell-specific aptamers have been conjugated with therapeutic agents and delivery vehicles, including small chemical drugs, oligonucleotides, and nanocarriers, for targeted delivery [4,172,173]. In this section, we discuss studies published during the past five years that have used aptamers as cancer-targeting agents.

### 6.1. Aptamer–Small Compound Conjugates

**Figure 3:**
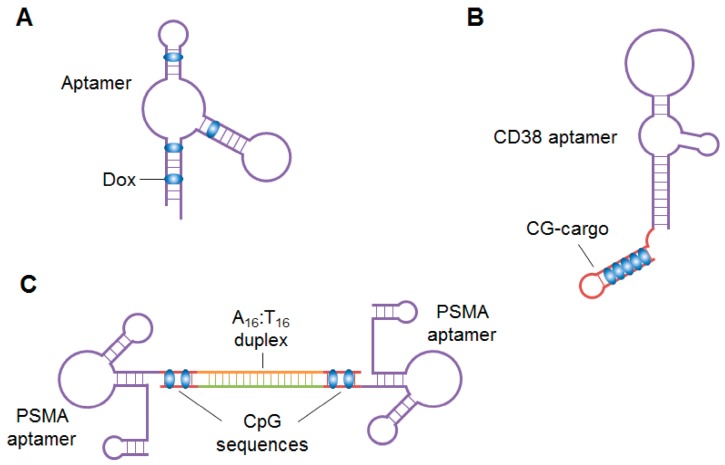
Schematics of aptamer-drug conjugates. (**A**) Non-covalent conjugation between an aptamer and Dox via intercalation. Dox is intercalated into the GC-rich region of the aptamer; (**B**) Aptamer-Dox conjugate with a CG-cargo structure. The CD38 aptamer is non-covalently conjugated to Dox via a CG-cargo structure. The aptamer–Dox conjugate forms at a 1:5 molar ratio of aptamer to Dox; (**C**) Dimeric aptamer complex bound to Dox. The aptamer complex consists of two PSAM aptamers dimerized via the A_16_ and T_16_ tails at their 3′-temini. Dox is covalently conjugated to the CpG sequences in the aptamers.

Aptamer–drug conjugates are especially useful for chemotherapeutic agents that have systemic side effects. Dox, a traditional chemotherapeutic agent that induces cancer cell death by intercalating into DNA, has been used as a model agent for cell-specific aptamer conjugation. Some groups demonstrated that Dox can non-covalently conjugate to aptamers, via intercalation into their GC-rich regions (Figure 3A), for delivery into specific cells [174,175,176,177]. Over the past five years, several other groups have reported novel types of aptamer–Dox conjugates. Wen et al. isolated a CD38-targeting DNA aptamer and non-covalently conjugated Dox to it in a CG-repeat structure, termed CG-cargo (Figure 3B) [178]. Using the CG-repeat structure, the aptamer-Dox conjugate formed with a 1:5 molar ratio of aptamer to Dox. When systemically administered to multiple myeloma-bearing mice, the conjugate specifically released Dox in tumor cells, inhibited tumor growth and improved survival rates of mice. CG-cargo can carry a high payload of Dox and may be conjugated to other aptamers to target different cancers. Trinh et al. generated a drug-DNA adduct called AS1411-Dox by crosslinking Dox and AS1411 with formaldehyde at 10 degrees overnight [179]. When systemically injected into hepatocellular carcinoma-bearing mice, AS1411-Dox inhibited tumor growth without causing severe toxicity to non-tumor tissues. Generation of the adduct was simply and cheap, suggesting that it may be widely used, particularly in developing counties, to produce other aptamer-Dox conjugates that will reduce the systemic toxicity of Dox. To further improve the targeting ability of monovalent PSMA aptamer-Dox conjugates, Boyacioglu et al. developed a dimeric PSMA aptamer complex (DAC) bound to Dox (Figure 3C) [180]. The PSMA aptamers were synthesized with either A_16_ or T_16_ tails at their 3′-temini. DACs were prepared by mixing the A_16_- and T_16_-tailed PSMA aptamers at a 1:1 ratio. Dox was covalently conjugated to the CpG sequences in DACs through a pH-sensitive linker, so the DAC-Dox conjugates were stable under physiological conditions but dissociated after internalization into PSMA-positive cells. As a result, the DAC conjugate specifically inhibited growth of PSMA-positive cells. In addition to enhancing Dox-induced therapeutic toxicity to the targeted cancer cells, DACs may improve the pharmacokinetic properties (e.g., circulation time and half-life) of aptamer-Dox conjugates in vivo due to their increased molecular weight. 

Covalent conjugation to aptamers has also been used to target other chemotherapy agents to cancer cells. For example, Zhao et al. developed a cell-specific aptamer—methotrexate (MTX) conjugate to specifically inhibit AML [181]. They first isolated a DNA aptamer targeting CD117, which is highly expressed on AML cells. The DNA aptamer, which contains a G-quadruplex structure, was covalently conjugated with MTX via amine coupling reaction using N-hydroxysuccinimide (NHS). The CD117 aptamer-MTX conjugate specifically inhibited AML cell growth.

### 6.2. Aptamer-Therapeutic Oligonucleotide Conjugates

Like aptamers, several other types of oligonucleotides, including small interfering RNA (siRNA), micro RNA (miRNA), and anti-miRNA (antimiR), are attractive as therapeutic agents because they can modulate the expression of specific cancer targets, including undruggable oncogenes that could not be targeted pharmacologically [182]. Because these oligonucleotides function inside cells, it is important to deliver them efficiently. Since the first report of PSMA aptamer-siRNA chimeras in 2006 [183], many aptamer-oligonucleotide conjugates have been developed for anticancer therapy [4,105,184]. Among the various therapeutic oligonucleotide conjugates, siRNA conjugates are the most popular.

**Figure 4:**
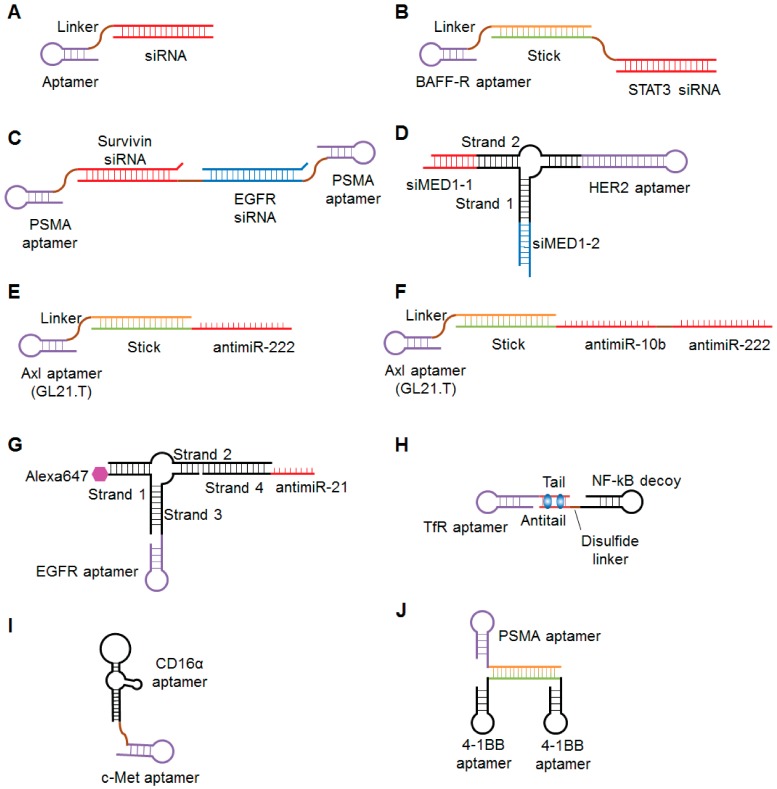
Schematic of aptamer-oligonucleotide conjugates. (**A**) Aptamer-siRNA chimera. An EpCAM aptamer is covalently linked to a PLK1-specific siRNA sense strand; (**B**) Aptamer–stick–siRNA conjugates. One of the two STAT3 siRNA strands is linked to the 3′ end of a BAFF-R aptamer through a “sticky bridge” sequence; (**C**) Bivalent PSMA aptamer-dual siRNA chimera. Two PSMA aptamers flank siRNAs specific to survivin and EGFR; (**D**) Three-way junction pRNA nanoparticle. pRNA-HER2apt-siMED1 consists of a HER2-targeting RNA aptamer and two different siRNAs targeting MED1 connected by a three-way junction pRNA; (**E**) Aptamer-antimiR conjugates. The aptamer GL21.T is non-covalently conjugated to antimiR-222 through a stick sequence; (**F**) Aptamer-dual antimiR conjugate. GL21.T is non-covalently conjugated to antimiR-10b and antimiR-222 through a stick sequence; (**G**) Three-way junction-EGFR aptamer-antimiR-21 nanoparticle. The nanoparticle consists of four strands bearing an EGFR aptamer, Alexa647 and antimiR-21; (**H**) Aptamer-decoy-Dox complex. The 3′ end of the anti-transferrin receptor RNA aptamer is elongated with a short DNA tail (CGA)_7_ complementary to a DNA anti-tail (TCG)_7_ that is conjugated to the 3′ end of the NF-κB decoy by a disulfide linker. The GC-rich region in tail/anti-tail is a putative Dox binding site; (**I**) Bi-specific aptamers as a cell engager. CD16α specific DNA aptamer is covalently conjugated to c-Met aptamer; (**J**) PSMA aptamer-dimeric 4-1BB aptamer conjugates. A PSMA aptamer is non-covalently conjugated to a dimeric 4-1BB aptamer.

Recently, several groups demonstrated that EpCAM-targeting aptamers are good tools for siRNA delivery into epithelial cancers and cancer stem cells. EpCAM is a tumor-associated antigen that is highly expressed on epithelial cancers and their associated cancer stem cells [185]. Subramanian et al. reported very strong tumor regression after injection of an EpCAM-targeting aptamer- siRNA conjugates in an MCF-7 epithelial cancer xenograft model [186]. EpCAM-AsiC is an EpCAM aptamer covalently linked to a polo-like kinase (PLK1)-specific siRNA sense strand annealed to its antisense strand (Figure 4A). Gilboa-Geffen et al. showed that EpCAMiC is specifically taken up by EpCAM-positive cancer cell lines and in human EpCAM-positive breast cancer biopsies, where it silences the expression of PLK1 [187]. EpCAM-AsiC subcutaneously administered at 5 mg/kg every three days for two weeks suppressed cancer growth in a EpCAM-positive TNBC xenograft model. Survivin, a member of the inhibitor of apoptosis (IAP) protein family that inhibits caspases and blocks cell death, is overexpressed in the cancer stem cell population of Dox-resistant breast cancer cells. Wang et al. demonstrated that an EpCAM aptamer-survivin siRNA chimera that specifically targeted cancer stem cells in a mouse xenograft model induced survivin knockdown, which therefore resulted in the reversal of Dox resistance [188]. Collectively, the EpCAM aptamers have proven their application for effective delivery of anticancer agents and reversal of chemoresistance for killing cancer stem cells.

To enhance the pharmacological efficacy of siRNA-based anticancer therapeutics, multi-functional and multi-targeting strategies have recently been used. In 2013, Zhou et al. developed dual-functional B-cell-activating factor receptor (BAFF-R) aptamer-siRNA conjugates for B-cell malignancies [189]. They isolated several BAFF-R aptamers that could efficiently bind to BAFF-R on the surface of B-cells and compete with BAFF to inhibit BAFF-mediated B-cell proliferation. They further conjugated the inhibitory BAFF-R aptamer with siRNA against human STAT3 for achieving a dual inhibitory effect. STAT3 plays an important role in promoting the progression of human cancers including several types of B-cell lymphoma. Two different types of BAFF-R aptamer-STAT3 siRNA conjugates, a covalent aptamer-siRNA chimera and a non-covalent aptamer-stick-siRNA conjugate (Figure 4B), blocked BAFF-mediated signaling and specifically decreased the expression of STAT3 in human B-cell lines. Multi-functional aptamer-siRNA conjugates, in which both the aptamer and siRNA can suppress their corresponding targets, may be more effective than single-function conjugates at controlling tumor progression. More recently, Liu et al. reported a bivalent aptamer-dual siRNA chimera for prostate cancer (Figure 4C) [190]. This aptamer-siRNA chimera consists of two PSMA aptamers and two siRNAs targeting EGFR and survivin. The chimera reduced the expression of EGFR and survivin, induced apoptosis, and effectively suppressed tumor growth and angiogenesis in a prostate cancer xenograft model. Compared to monovalent conjugates in which only one aptamer delivers one siRNA, bivalent conjugates improve aptamer-mediated targeting avidity and increase siRNA cargoes for improved gene silencing. Zhang et al. developed RNA nanoparticles containing a three-way junction motif derived from bacteriophage phi29 packaging RNA (pRNA) bearing a HER2-targeting RNA aptamer and two different siRNAs targeting estrogen receptor coactivator Mediator Subunit 1 (MED1) to overcome tamoxifen-resistant breast cancer [191]. These multi-functional nanoparticles (pRNA-HER2apt-siMED1) (Figure 4D) specifically bound to HER2-positive cells and inhibited MED1 expression and cell growth. More importantly, pRNA-HER2apt-siMED efficiently reduced the growth and metastasis of breast cancer cells and sensitized them to tamoxifen treatment after systemic administration in a xenograft mouse model. These pRNA-based nanoparticles were stable and exhibited a favorable pharmacokinetic profile with multi-functional properties, such as targeted co-delivery of various therapeutics, suggesting feasible translation for clinical use.

In addition to delivering anticancer therapeutics, aptamer-siRNA conjugates have potential roles in cancer immunotherapy. Cytotoxic T lymphocyte-associated antigen 4 (CTLA4) is a cell surface receptor that functions as an immune checkpoint and downregulates immune responses [192]. Herrmann et al. developed a CTLA4 aptamer-STAT3 siRNA conjugate [193]. When locally or systemically administered, this conjugate significantly reduced the number of tumor-associated regulatory T cells and inhibited the growth of various tumors, including melanoma, renal cell carcinoma, colon carcinoma and human T cell lymphoma, in mice. More recently, Rajagopalan et al. showed that a 4-1BB aptamer-CD25 siRNA conjugate has potential utility for cancer immunotherapy [194]. 4-1BB is a major immune stimulatory receptor expressed on the surface of CD8+ T cells [195], and CD25 is the alpha subunit of the Il-2 receptor that has an important role in the differentiation of CD8+ T cells [196]. Intravenous injection of the 4-1 BB aptamer-CD25 siRNA conjugate reduced CD25 expression and downregulated IL-2 signaling in circulating CD8+ T cells in mice. Furthermore, administration of this conjugate improved the antitumor activity induced by vaccines and radiation.

Similarly to siRNA, other types of oligonucleotides, such as miRNA, antimiR, small activating RNA (saRNA), and decoy DNA, have been conjugated to cell-specific aptamers. The tyrosine kinase receptor Axl that is overexpressed in several human cancers is closely related to invasiveness and therapeutic resistance [197]. Recently, an RNA aptamer, GL21.T has been identified to specifically bind to and antagonize the Axl [198]. Recent studies have demonstrated the flexible use of this aptamer in aptamer-therapeutic oligonucleotide conjugates. Esposito et al. developed a dual-functioning aptamer-miRNA conjugate termed GL21.T-let [199]. The conjugate consists of GL21.T and the anti-oncogenic miRNA let-7g. When systemically injected into mice bearing Axl-positive or -negative lung cancer, GL21.T-let specifically delivered let-7g into Axl-positive tumors and inhibited their growth, but not inhibit the growth of Axl-negative tumors. Iaboni et al. demonstrated that GL21.T-conjugated miR-212 enhances sensitization to TNF-related apoptosis-inducing ligand (TRAIL) in human non-small cell lung cancer cells [200]. Catuogno et al. demonstrated that GL21.T can deliver antimiRs, as well [201]. They non-covalently conjugated GL21.T to the tumor suppressing antimiR-222 through a stick sequence (Figure 4E). This conjugate showed synergistic inhibition of cell migration and enhanced sensitivity to temozolomide (TMZ) treatment of Axl-positive cells. To further enhance the therapeutic potential of aptamer-antimiR conjugates, the same group non-covalently conjugated GL21.T to two antimiRs, antimiR-222 and antimiR-10b, which target corresponding oncomiR, respectively (Figure 4F). This multi-functional conjugate reduced the expression of both miR-222 and miR-10b. Esposito et al. studied a combination of aptamer-miRNA and aptamer-antimiR conjugates [202]. They used GL21.T and a platelet-derived growth factor receptor aptamer (Gint4.T) as carriers for miR-137 and antimiR-10b, respectively, and demonstrated that these conjugates synergize to inhibit the growth and migration of glioblastoma stem-like cells. Shu et al. conjugated antimiR-21 and Alexa-647 dye to an EGFR aptamer in a three-way junction pRNA (Figure 4G) [203]. When intravenously administration to TNBC-bearing mice, the RNA complexes were specifically delivered to TNBC cells, where they reduced the expression of miR-21, increased the expression of downstream target mRNAs, and inhibited tumor growth.

saRNA is a novel type of small double-stranded RNA that targets specific sequences in promoter regions to upregulate the expression of target genes. Downregulation or mutation of CCAAT/enhancer-binding protein-α (C/EBPα) has been reported to associate with tumor aggressiveness [204], suggesting it may be an important target for cancer, such as liver cancer and pancreatic cancer. Yoon et al. isolated two RNA aptamers that specifically target pancreatic adenocarcinoma cells and conjugated them to an saRNA targeting C/EBPα [205]. Both conjugates induced expression of C/EBPα, inhibited cell proliferation in pancreatic adenocarcinoma cell lines. These conjugates also reduced tumor growth when systemically administered to human pancreas cancer xenografts.

Porciani et al. combined the concepts of aptamer-small drug and aptamer-oligonucleotide conjugates [206]. They developed an RNA aptamer-DNA decoy chimera and loaded Dox into the GC-rich region. The chimera consisted of an RNA aptamer targeting the transferrin receptor and a DNA decoy for nuclear factor κB (NF-κB) DNA (Figure 4H). Using a secondary structure prediction tool, six to seven Dox molecules were assumed to bind to the GC-rich region in the tail/antitail. This multiple conjugate was effectively internalized and released Dox in tumor cells. Moreover, the conjugate inhibited NF-κB activity and enhanced Dox-induced apoptosis in pancreatic tumor cells.

The therapeutic payload can also be nucleic acid aptamer, therefore resulting in bi-specific aptamer conjugates for cancer immunotherapy with increased targeting and specificity [207,208,209]. For example, Boltz et al. isolated CD16α specific DNA aptamers which is able to specifically recognize natural killer (NK) cells. By rationally conjugating CD16α aptamer with a c-Met aptamer they generated bi-specific aptamer conjugates (Figure 4I) [210]. The bi-specific aptamer conjugated simultaneously bound to CD16α expressing NK cells and c-Met-overexpressing tumor cells, which specifically recruited NK cells to tumor cells, consequently inducing tumor cell lysis. In another study, Gilboa et al. develop a bivalent 4-1BB aptamers to function as an agonist, therefore promoting tumor immunity in mice. 4-1BB is a major co-stimulatory receptor expressed on activated CD8+ T cells. Because non-targeting activation may cause some unwanted toxicities, the same group constructed a PSMA aptamer- dimeric 4-1BB aptamer conjugates (Figure 4J) [211]. Compared to 4-1BB antibodies or non-targeting dimeric 4-1BB aptamers, the resulted bi-specific aptamer conjugates effectively induced co-stimulation at the PSMA-expressing tumor site at a reduced dosage. Taken together, these studies suggest that aptamers hold great promise for cancer immunotherapy.

### 6.3. Aptamer-Related Nanoparticles

In addition to delivering directly conjugated therapeutic drugs, aptamers can be used to deliver and functionalize nanomaterials, including liposomes, AuNPs, polymers, and dendrimers. 

**Figure 5:**
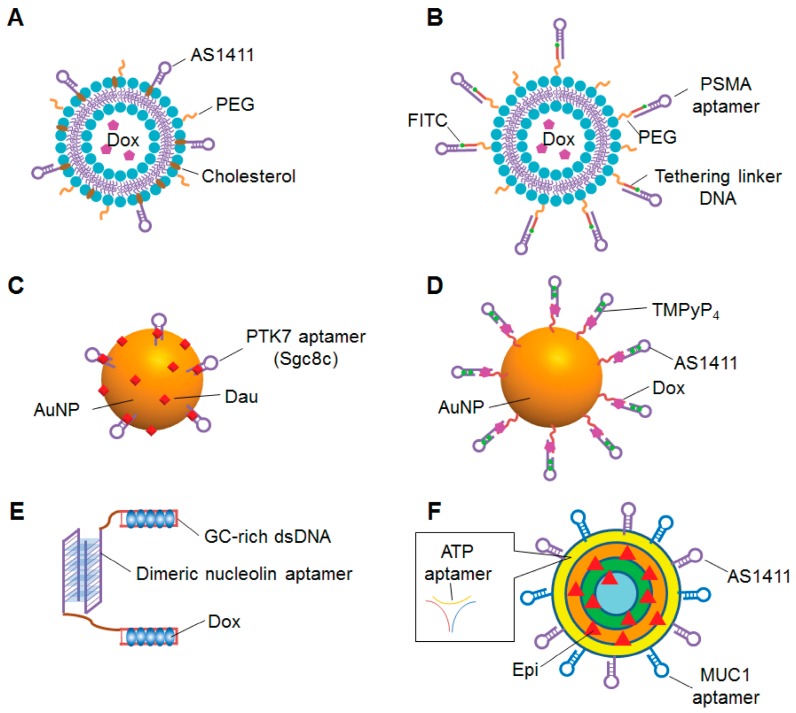
Schematics of aptamer-decorated nanocarriers. (**A**) AS1411-conjugated liposome. Cholesterol-modified nucleolin aptamers (AS1411) are immobilized onto the surface of a PEGylated liposome; (**B**) An aptamosome. A PSMA aptamer is conjugated to a PEGylated liposome by annealing to linker DNA modified with FITC and covalently conjugated to the termini of PEG; (**C**) Apt-Dau-AuNP complex. A PTK7-targeting aptamer (sgc8c) is conjugated to AuNPs simply by mixing them. Dau is loaded onto the surface of the AuNP and intercalated into the sgc8c aptamers; (**D**) Co-drug-loaded aptamer-conjugated AuNP. AS1411 is extended with a 27-base T6(CGATCGA)3 sequence at the 3′ end. After hybridization with the complementary sequence 5′ thiol-T10(TCGATCG)3, the double stranded AS1411 is immobilized onto the surface of AuNP. Dox molecules are loaded onto the CG-rich region within the extended sequence. The photosensitizer TMPyP4 is non-covalently attached to the AS1411-conjugated AuNP; (**E**) Aptamer-dsDNA and Dox nanoparticles. The aptamer part forms a dimeric G-quadruplex nanostructure. The dsDNA consists of GC-rich region to deliver the Dox payload; (**F**) Apts-Dendrimer-Epi complex. The DNA-dendrimer is prepared by mixing several ssDNAs containing ATP aptamers. Epi is loaded onto the dendrimer by mixing, and MUC1 and AS1411 aptamers are non-covalently conjugated to the dendrimer-Epi encapsulate.

Among these nanomaterials, liposomes are promising carriers for anticancer chemotherapeutic agents because they specifically target tumor tissue and reduce the systemic toxicities of anticancer drugs [212]. Doxil is a PEGylated liposomal Dox (PL-Dox) that is approved for clinical use by the US Food and Drug Administration [213]. Although Doxil effectively reduces the side effects of Dox, there is still room to improve drug efficacy because Doxil is passively targeted to tumors lacks a targeting agent. Recently, several groups reported aptamer conjugation techniques for targeted therapy of PL-Dox. Xing et al. attached the nucleolin-targeting aptamer AS1411 with PL-Dox (Figure 5A) [214]. As compared to a non-targeting liposome, the AS1411-conjugated liposome increased cellular uptake and cytotoxicity of Dox in breast cancer cells. Furthermore, when intratumorally injected into breast-cancer-xenograft mice, the AS1411-conjugated liposome enhanced the antitumor efficacy of Dox compared to a non-targeting liposome. The same group also optimized the length and composition of spacer molecules on the surface of AS1411-conjugated liposomes, and showed that spacer length is an important factor for the targeting ability of the AS1411 aptamer [215]. Baek et al. conjugated a PSMA-specific RNA aptamer to a PEGylated liposome, and called it an “aptamosome” (Figure 5B) [216]. A Dox-encapsulating aptamosome showed specific binding to, uptake by, and cytotoxicity against PSMA-positive prostate cancer cells. When intravenously administrated to prostate-cancer-xenograft mice, the Dox-encapsulating aptamosomes specifically accumulated in, were retained by, and inhibited the growth of tumor tissues. Moosavian et al. conjugated TSA14, an RNA aptamer that specifically targets breast cancer cells, to the surface of PL-Dox [217]. Compared to aptamer- unmodified PL-Dox, aptamer-conjugated PL-Dox showed improved cellular uptake and cytotoxicity in breast cancer cell lines. When intravenously injected into breast cancer xenograft mice, aptamer-conjugated PL-Dox accumulated in tumor tissue and enhanced inhibition of tumor growth compared to non-targeting liposomes.

Zhen et al. developed an aptamer-conjugated liposome for the CRISPR/Cas9 system [218]. They encapsulated a PLK1-targeting CRISPR/Cas9-gRNA into a PSMA aptamer (A10)-conjugated liposome. The A10-liposome-CRISPR/Cas9 chimera showed specific binding to and uptake by PSMA-positive cells and enhanced PLK1 knockdown and cell apoptosis. When intravenously injected into mice bearing PSMA-positive prostate cancer, the A10-liposome-CRISPR/Cas9 chimera significantly reduced tumor size.

Recent studies have also focused on AuNPs as aptamer-functionalized carriers because of their favorable characteristics, such as biocompatibility, low toxicity, and large surface area for decoration. Over the past five years, several groups have reported sophisticated aptamer-AuNPs systems for cancer therapy. To enhance the tumor targeting of hollow gold nanospheres (HAuNS), Zhao et al. covalently conjugated HAuNS to RNA aptamers specific for CD30, a diagnostic biomarker for Hodgkin’s lymphoma and anaplastic large cell lymphoma [219]. The Dox-loaded aptamer-conjugated HAuNS (Apt-HAuNS-Dox) showed specific binding to CD30-positive lymphoma cells. In addition to selective binding, Apt-HAuNS-Dox effectively released the loaded Dox into the target cells and into a low pH solution similar to the lysosomal environment. Most importantly, this pH-sensitive Apt-HAuNS-Dox selectively killed the CD30-positive lymphoma cells in mixed cultures with CD30-negative cells. Danesh et al. reported the use of AuNPs conjugated to PTK7 aptamers for selective delivery of daunorubicin (Dau) to T cell acute lymphoblastic leukemia [220]. The PTK7-targeting aptamer, sgc8c, was conjugated to AuNPs, and Dau was loaded onto the surface of the AuNPs and intercalated into the sgc8c aptamers (Figure 5C). The Apt-Dau-AuNP complexes effectively released Dau in an acidic environment and showed selective internalization and cytotoxicity to the targeted T cell line. The same group enhanced the therapeutic efficacy of the Apt-Dau-AuNPs complex by using two kinds of aptamers, sgc8c and AS1411, to target cancer cells [221]. The polyvalent aptamer-conjugated AuNPs showed pH-dependent Dau release and selective internalization into target cells. As compared to the sgc8c aptamer-conjugated complex, the polyvalent Apt-Dau-AuNP complex was more cytotoxic to target cells and less cytotoxic to non-target cells.

Dual-targeting AuNPs was also developed by Chen et al. They conjugated gold nanoclusters with the nucleolin aptamer AS1411 and cyclic peptide RGD (cRGD), a ligand of the integrin alpha-v beta 3, which is overexpressed on the surface of tumor cells [222]. The dual-targeting AuNP (AuNC-cRGD-Apt) was functionalized with near infrared fluorescence dye for tumor imaging or Dox loading for tumor therapy. AuNC-cRGD-Apt was efficiently internalized and delivered Dox to nuclei in target cells. When systemically injected in malignant glioma xenograft mice, the dual-targeting AuNP accumulated in tumor tissue and inhibited tumor growth without causing severe toxicity in non-tumor tissues. In addition to multi-targeted delivery, aptamer-conjugated AuNPs have been developed for multidrug delivery as well. Shiao et al. non-covalently attached the photosensitizer 5,10,15,20-terakis(1-methlpyridinium-4-yl) porphyrin (TMPyP4) and Dox to AS1411-conjugated AuNPs (Figure 5D) [223]. When this co-drug complex was delivered into nucleolin-positive cells, light exposure induced production of reactive oxygen species, release of Dox, and synergistic cytotoxicity.

As mentioned above, the use of multi-functional aptamer-based nanoparticles is a potential strategy to enhance drug efficacy and reduce the side effects of cancer treatment. Recently, some groups have also developed multi-functional aptamer-based nanoparticles using oligonucleotides that can self-assemble into three-dimensional nanostructures. Liu et al. developed a multi-functional DNA nanostructure consisting of a dimeric nucleolin aptamer and GC-rich dsDNA for Dox delivery to resistant cancer cells (Figure 5E) [224]. Dox was efficiently intercalated into the GC-rich dsDNA region, and the DNA nanoparticles enhanced the Dox sensitivity of resistant breast cancer cells, perhaps in part by inducing S phase arrest of resistant breast cancer cells, increasing cellular uptake and decreasing efflux of Dox. When intravenously injected into Dox-resistant breast cancer xenograft model, the DNA complex strongly inhibited tumor growth and reduced cardiotoxicity compared to free Dox treatment. Taghdisi et al. attached three different aptamers targeting MUC1, nucleolin and ATP to a DNA dendrimer for targeted delivery of epirubicin (Epi) [225]. This Apts-Dendrimer-Epi complex (Figure 5F) was specifically internalized by and cytotoxic to target tumor cells. When intravenously injected in colon carcinoma xenograft mice, the Apts-Dendrimer-Epi complex strongly reduced tumor growth.

## 7. Conclusions and Perspective

Based on their unique characteristics, such as fast in vitro selection and facile chemical synthesis and site-specific modification, related small physic size, high thermal stability, and better tissue penetration, a number of aptamers have been developed as versatile tools for cancer imaging, diagnosis and therapy. In this review, we have focused on the progress of aptamer technology in the field of cancer over the past five years. During this time, the aptamer selection process has been improved to identify high-affinity target-specific aptamers, and high-throughput technologies will continue to reduce the time required for isolation of aptamers. To date, although only two aptamer-based cancer therapeutics have undergone clinical trials, several more aptamers have shown great potential for cancer imaging, diagnosis, and therapy. Use of covalent or non-covalent conjugation strategies allows different aptamers to serve as easily exchanged building blocks for functionalizing other therapeutic agents. This feature may greatly facilitate their clinical translation. In particular, multi-specific nucleic acid aptamer-based immunotherapeutic modality is being exploiting for engaging cancer-specific immunity to eliminate tumor cells. Although bi-specific antibodies, such as BiTE, are currently used for this purpose, there are several concerns with clinical translation of antibody-based immunotherapeutics, including significant autoimmune toxicities associated with repeat administration, and high cost and complexity associated with bi-specific antibody conjugate development and production in cGMP standard facilities. A nucleic acid aptamer-based platform is superior to current antibody-based strategies, as aptamers allow: (1) better tissue penetration; (2) lack of immunogenicity; (3) faster target accumulation and shortened body clearance, enabling the use of shorter-lived radioisotopes; (4) simpler, better controlled, and thus less expensive chemical production; (5) lack of aggregation issues; (6) amenability to a variety of chemical modifications that are needed for production and storage, such as pH changes or elevated temperature. Aptamers are becoming increasingly common as therapeutics; as of May 2016, there were ten aptamers investigated for clinical applications, and one had received FDA approval. Nevertheless, some practical hurdles, such as the high cost of modified oligonucleotides and insufficient survival in vivo due to nuclease-mediated degradation and rapid renal filtration, still hinder the development of aptamer-based tools. Addressing these challenges will improve the versatility of aptamers for cancer treatment in the future.

## Figures and Tables

**Table 1:** Summary of sandwich style-aptasensors and label-free aptasensors targeting cancer. S: sandwich-type aptasenser; LF: label-free aptasensor, LOD: limit of detection.

Cancer Type	Target	Group	Type	Reported Sensitivity (Linear Range)	LOD
Breast	HER2	Hu, 2017 [50]	S	0.01–5 ng/mL	5 pg/mL
		Shen, 2017 [51]	S	1–100 pg/mL	0.047 pg/mL
	MUC1	Zhang, 2017 [44]	S	5 × 10^2^–1 ×10^6^ cells/mL	1 × 10^2^
		Chen, 2015 [52]	S	1–100 nM	1 pM
		Zhu, 2013 [53]	S	100–10^7^ cells/mL	100 cells
	OPN	Meirinho, 2017 [54]	LF	25–100 nM	1 nM
	BRCA-1 sequence	Yang, 2014 [55]	LF	1 pM–500 nM	1 pM
Cervical	HeLa (Nucleolin)	Wang, 2015 [56]	LF	10–10^6^ cells/mL	10 cells/mL
Colorectal	CT26 cells	Hashkavayi, 2017 [57]	LF	10–10^5^ cells/mL	2 cells/mL
	HCT116 cells (CEA)	Ahmadzadeh-Raji, 2016 [58]	LF	1–25 cells/mL	6 cells/mL
Liver	HepG2 cells	Sun, 2016 [59,60]	S	10^2^–10^7^ cells/mL	15 cells/mL
		Kashefi-Kheyrabadi, 2014 [61]	LF	10^2^–10^6^ cells/mL	2 cells/mL
Leukemia	CCRF-CEM cells	Amouzadeh Tabrizi, 2017 [62]	S	10–5 × 10^5^ cells/mL	8 cells/mL
	HL-60, CEM cells	Zheng, 2013 [63]	S	5 × 10^2^–1 × 10^7^ cells/mL	350 cells/mL
Lung	VEGF	Amouzadeh Tabrizi, 2015 [64]	LF	10.0–300 pg/mL	1.0 pg/mL
		Shamsipu, 2015 [65]	LF	2.5–250 pM	0.48 pM
	Postop lung cancer tissue and CTCs	Zamay, 2016 [66]	S	10–100 ng/mL	0.023 ng/mL
Lymphoma	Ramos cells	Zhong, 2011 [67]	S	10–1000 cells	10 cells
		Yi [68]	LF	10–10^6^ cells	10 cells
Prostate	PSA	Zhu, 2016 [69]	S	0.05–500 fg/mL	0.2 fg/mL
		Heydari-Bafrooei, 2017 [49]	LF	0.005–20 ng/mL	1 pg/mL
		Tamboli, 2016 [70]	LF	0.1 pg/mL–1 ng/mL	0.1 pg/mL
		Tzouvadaki, 2016 [71]	LF		23 aM
		Kavosi, 2015 [72]	LF	0.1 pg/mL–90 ng/mL	10 fg/mL
		Souada, 2015 [73]	LF	1 ng/mL–10 ug/mL	
Multiple	CEA	Wang, 2016 [74]	S	5 fM–500 nM	5 fM
	EGFR	Ilkhani, 2015 [75]	S	1–40 ng/mL	50 pg/mL
	EpCAM	Yan, 2013 [76]	S	100–5 × 10^7^ cells/mL	38 cells/mL
	Micro RNA mIR-21	Wang, 2016 [77]	LF	1 fM–10 nM	0.75 fM

**Table 2:** Aptamers for cancer therapy in clinical trials.

Drug Name and Company	Target	Form and Modification	Sequence	Target Disease	Administration Route	ClinicalTrials.gov Identifier(Condition, Current Status)
AS1411 (AGRO-100) Antisoma Advanced Cancer Therapeutics	Nucleolin	26-mer unmodified guanosine-rich oligonucleotide PEGylated	5′-GGTGGTGGTGGTTGTGGTGGTGGTGG-3′	metastatic renal cell carcinoma relapsed or refractory AML	intravenous	NCT00881244 (Advanced Solid Tumors, phase I, completed) NCT00740441 (Metastatic Renal Cell Carcinoma, phase II, unknown) NCT01034410 (AML, phase II, terminated) NCT00512083 (Leukemia, Myeloid, phase II, completed)
NOX-A12 (Olaptesed pegol) NOXXON Pharma	CXCL12	45-mer l-ribonucleic acid (Spiegelmer) PEGylated	5′-CGCAUGGACUGAUCCUAGUCGGUUAUGUAGAUCUAGUGUGGUGCG-3′	multiple myeloma chronic lymphocytic leukemia autologous stem cell transplantation hematopoietic stem cell transplantation metastatic colorectal cancer metastatic pancreatic cancer	intravenous	NCT01521533 (Multiple Myeloma, phase II, completed) NCT01486797 (Chronic Lymphocytic Leukemia, phase II, completed) NCT00976378 (Autologous Stem Cell Transplantation, phase I, completed) NCT01194934 (Hematopoietic Stem Cell Transplantation, phase I, completed) NCT03168139 (Metastatic Colorectal Cancer, phase I, Metastatic Pancreatic Cancer, phase II, recruiting)
